# 高碳含量新型亚微米无孔二氧化硅材料的修饰方法及其在反相加压毛细管电色谱平台上的应用

**DOI:** 10.3724/SP.J.1123.2021.03042

**Published:** 2022-01-08

**Authors:** Zihang XIA, CHEDDAH Soumia, Weiwei WANG, Yan WANG, Chao YAN

**Affiliations:** 上海交通大学药学院, 上海 200240; School of Pharmacy, Shanghai Jiao Tong University, Shanghai 200240, China; 上海交通大学药学院, 上海 200240; School of Pharmacy, Shanghai Jiao Tong University, Shanghai 200240, China; 上海交通大学药学院, 上海 200240; School of Pharmacy, Shanghai Jiao Tong University, Shanghai 200240, China; 上海交通大学药学院, 上海 200240; School of Pharmacy, Shanghai Jiao Tong University, Shanghai 200240, China; 上海交通大学药学院, 上海 200240; School of Pharmacy, Shanghai Jiao Tong University, Shanghai 200240, China

**Keywords:** 加压毛细管电色谱, 亚微米无孔二氧化硅微球, 高碳含量, 硅羟基, 电渗流, pressurized capillary electrochromatography (pCEC), submicron nonporous silica microsphere, high carbon content, silanol groups, electroosmotic flow (EOF)

## Abstract

亚微米无孔二氧化硅(NPS)材料具有小粒径及表面光滑形状规整等特点,是一种性能优异的色谱材料,但其存在比表面积小、修饰效率低的问题。针对此设计了一种具有高碳含量的修饰方法:以3-缩水甘油基氧基丙基三甲氧基硅烷(GPTS)作为硅烷偶联剂,聚乙烯亚胺(PEI)作为聚合物包覆层,并以硬脂酰氯修饰得到一种氨基包覆的具有C_18_碳链结构的新型亚微米无孔二氧化硅材料(C_18_-NH_2_-GPTS-SiO_2_)。利用元素分析、傅里叶变换红外光谱、Zeta电势等进行表征,证明C_18_-NH_2_-GPTS-SiO_2_固定相的成功制备。该修饰方法将NPS的碳含量从0.55%提高到了8.29%,解决了过往NPS材料采用十八烷基氯硅烷等传统C_18_修饰方法时碳含量较低的问题。此外,^29^Si固体核磁显示:NPS与多孔二氧化硅(PS)微球相比不仅存在孔结构与比表面积区别,且表面硅羟基种类也不同。16%的PS微球硅原子带有一个硅羟基(孤立硅羟基,Q_3_)、19%带有两个硅羟基(偕硅羟基,Q_2_);而NPS微球不存在偕硅羟基,仅有30%硅原子处于孤立硅羟基状态。实验发现NPS微球存在硅羟基数量低且缺少偕硅羟基的特点,导致NPS材料表面修饰活性低,难以通过简单一步反应获得高碳含量。采用不同疏水物质如苯系物、多环芳烃对色谱性能及保留机理进行研究,结果表明C_18_-NH_2_-GPTS-SiO_2_色谱柱符合反相作用机理。氨基的包覆改变硅球表面电性,提高了NPS材料运用于加压毛细管电色谱平台(pCEC)时的电渗流大小,施加+15 kv时,显示出良好的分离能力,证实了C_18_-NH_2_-GPTS-SiO_2_材料通过多步反应提高碳含量的修饰方法在pCEC平台上应用的优异性。

## Abstract (en)

Submicron nonporous silica (NPS) materials feature small particle sizes, smooth surfaces, and regular shapes. They also exhibit excellent performance as a stationary phase; however, their use is limited by their low specific surface area and low phase ratio. Therefore, a novel surface modification strategy tailored for NPS microspheres was designed, involving a multi-step reaction. 3-Glycidyloxypropyltrimethoxysilane (GPTS) was first grafted onto NPS particles as a silane coupling agent. Polyethyleneimine (PEI), a high-molecular-weight polymer, was then coated onto the particles, providing numerous amino reaction sites. In the final step, an acylation reaction was initiated between stearoyl chloride and the amino groups to obtain the final product, designated as C_18_-NH_2_-GPTS-SiO_2_. Elemental analysis, FT-IR spectroscopy, Zeta potential analysis, thermogravimetric analysis (TGA), and scanning electron microscopy (SEM) were employed to investigate the success of the chemical modifications at each step. The carbon content increased from 0.55% to higher than 8.29%. Thus, it solved the low carbon loading capacity problem when modifying NPS microspheres with traditional C_18_ reversed phase (e. g., octadecyl chlorosilane modification).

Meanwhile, the reasons for the considerable differences between NPS and porous silica (PS) microspheres in terms of the reactivity to surface modification were investigated in detail. The BET method was employed to compare the pore structures. FT-IR and ^29^Si solid-state NMR spectroscopy were employed to analyze the differences in the structure and quantity of silanol groups on the surfaces of the NPS and PS microspheres. Differences were observed not only in the pore size and surface area, but also in the types of silanol groups. FT-IR analysis indicated that the NPS and PS microspheres had different *υ*_Si-OH_ band positions, which shifted from 955 to 975 cm^-1^, respectively. ^29^Si solid-state NMR analysis further highlighted the differences in structural information for Si atom environments. Results revealed that 16% of silicon atoms in the PS microspheres had one hydroxyl group (isolated silanols, Q_3_, *δ* 100), while 19% had two hydroxyl groups (geminal silanols, Q_2_, *δ* 90). On the other hand, the NPS microspheres possessed no geminal silanols, and only 30% of the Si atoms were in the Q_3_ state. Therefore, the NPS microspheres had a lower density of silanol groups and lacked geminal silanol groups, compared to the PS microspheres. Geminal silanol groups have already been confirmed in previous studies to offer far higher reactivity than isolated silanols. These factors together explained the low reactivity of NPS microspheres toward surface modification. Further, the low specific surface area of the microspheres arising from their nonporous nature made it challenging to obtain a high carbon content through a simple one-step reaction.

Hydrophobic substances such as hydrocarbons from the benzene series and polycyclic aromatic hydrocarbons (PAHs) were selected to study the chromatographic performance. The hydrophobic mechanism was revealed by the separation of PAHs using different ratios of acetonitrile. Separation was achieved with a C_18_-NH_2_-GPTS-SiO_2_ column, following which a hydrophobic phenomenon occurred. The presence of the amino coating led to the inversion of the electroosmotic flow (EOF) of the silica microspheres on the pressurized capillary electrochromatography (pCEC) platform. It also enhanced the linear velocity in the pCEC platform when the pH was selected to be low. The effects of the applied voltage on the separation ability of the 720 nm C_18_-NH_2_-GPTS-SiO_2_ column were examined to determine optimal conditions. Rapid and effective separation was achieved in a relatively short time. Therefore, the C_18_-NH_2_-GPTS-SiO_2_ stationary phase is promising for practical use with a higher phase ratio, demonstrating superiority for use in reversed-phase pCEC separation, and thus, providing a new strategy and valuable reference for the future application of submicron NPS microspheres.

随着液相色谱技术的发展,色谱填料正向着更小更均匀的方向发展^[1]^,亚微米无孔二氧化硅(NPS)材料开始逐渐被应用于毛细管填充柱与加压毛细管电色谱(pCEC)平台^[2,3]^。依据Van Deemter方程,亚微米级别粒径的填料能大幅提升色谱性能,且无孔材料相比多孔材料而言,不存在流动相在介孔结构中停滞流动从而造成传质阻力的问题,传质阻力减小,理论塔板高度进一步降低^[4]^。因此采用Stöber法^[5]^制备的亚微米NPS微球,具有小粒径及表面无孔的双重优势,是色谱理论上效能较高的色谱材料之一^[6]^。

但另一方面,亚微米NPS微球的比表面积较低^[7]^,当采用十八烷基氯硅烷等传统C_18_修饰方法修饰时,修饰效率低,碳含量通常仅为1%左右,远低于多孔材料的10%~17%^[4]^。在反相色谱分离机理下,分离效果不佳。因而需要发展亚微米NPS色谱填料的表面修饰新方法,增加表面碳含量,提高反相色谱的分离效率。比如采用树枝状的分子结构与聚合物表面包覆等方法可以巧妙地提高硅球表面的反应位点,通过多步反应的方式来提高碳含量。Niu等^[8]^将聚合物PEG-600包覆在亚2 μm NPS微球表面,在蛋白质样品中表现出良好的分析能力;Chu等^[9]^在5 μm二氧化硅颗粒表面,修饰大量的树枝状结构,通过发散状的方式循环反应获得了25.8%的高碳含量,并将其用作超高压液相色谱的新型填料。

二氧化硅微球表面硅羟基的数量与状态对二氧化硅球的分散性、稳定性及表面修饰活性等性质均有较大的影响^[10]^。本文首先对比NPS材料与多孔二氧化硅(PS)材料表面硅羟基的结构差异,在此基础上针对亚微米NPS微球设计一种表面修饰的新方法,将聚乙烯亚胺(PEI)包覆在二氧化硅微球表面,利用其自身大量的氨基作为反应位点,通过硬脂酰氯的酰基化反应得到含较高密度C_18_的新型亚微米反相NPS材料。采用傅里叶变换红外光谱、Zeta电势、元素分析、热重实验等手段对修饰方法进行表征;并利用pCEC平台可规避反压限制的特性^[11,12]^,将该亚微米级别的色谱填料运用于pCEC平台,对电渗流(EOF)的大小进行测定,并用苯系物及多环芳烃等物质考察其作用机理,证明本研究合成的以C_18_、氨基为特征,3-缩水甘油基氧基丙基三甲氧基硅烷(GPTS)连接的亚微米无孔二氧化硅固定相(C_18_-NH_2_-GPTS-SiO_2_)是一种与加压毛细管电色谱平台具有良好契合度的新型色谱填料。

## 1 实验部分

### 1.1 仪器、试剂与材料

TriSep-3000加压毛细管电色谱仪(美国Unimicro Technologies Pleasanton CA公司);熔融石英毛细管(100 μm i.d.,河北永年锐沣色谱器件有限公司);纳米粒度Zeta电位仪(美国Brookhaven公司);比表面积与孔隙度分析仪(美国Quantachrome仪器公司);红外光谱仪(美国Thermo Fisher公司); 600 MHz固体核磁共振波谱仪(瑞士Bruker公司); S-4800场发射扫描电子显微镜(日本Hitachi电子株式会社); Vario EL Cube型元素分析仪(德国Elementar公司); Tyris 1型热重分析仪(TGA, 美国PerkinElmer公司)。

720 nm NPS微球(美国Fiber Optic Center公司); 3 μm PS微球(加拿大Silicycle公司); GPTS (纯度97%)购自北京伊诺凯科技有限公司;聚乙烯亚胺(*M*_r_ 70000, 50%水溶液)购自上海侨怡生物科技有限公司;硬脂酰氯(纯度97%)购自上海麦克林生化科技有限公司;苯、甲苯、乙基苯、正丙基苯、正丁基苯、环己基苯、萘、苊、蒽、苯并[*a*]蒽、甲酸铵、甲酸均购自阿拉丁上海有限公司,纯度均不小于98%;乙腈(色谱纯)购于美国Tedia公司;浓硝酸、甲苯、丙酮、无水乙醚、无水乙醇、二氯甲烷等均为化学纯,购自国药集团化学试剂有限公司。

### 1.2 二氧化硅微球表面结构修饰

720 nm NPS微球的活化:称取2 g高温煅烧后的720 nm NPS微球于150 mL圆底烧瓶中,加入40 mL超纯水充分振荡超声后置于油浴锅中,加入40 mL浓硝酸,于140 ℃冷凝回流反应4 h。待反应结束后,将材料使用超纯水洗涤3次至洗涤液中性,无水乙醇洗涤2次后回收微球材料置于60 ℃真空干燥箱中干燥12 h,将材料标记为720 nm-activated-SiO_2_。

720 nm-activated-SiO_2_修饰环氧丙基:称取1.5 g上述反应制备的720 nm-activated-SiO_2_,于120 ℃真空干燥箱中除水6 h,随后转移至150 mL双颈烧瓶中,加入60 mL除水甲苯后,采用橡胶塞封口,充分振荡超声后置于油浴锅中,安装冷凝管及氮气保护装置后使用注射器从橡胶塞口注入9 mL GPTS,于110 ℃冷凝回流反应24 h。待反应结束后,将材料依次使用50 mL甲苯、丙酮、无水乙醚洗涤,回收反应后硅球材料置于60 ℃真空干燥箱中干燥12 h,备用,将材料标记为720 nm-GPTS-SiO_2_。

720 nm-GPTS-SiO_2_修饰聚乙烯亚胺:称取1 g上述反应制备的720 nm-GPTS-SiO_2_,于150 mL圆底烧瓶中,加入40 mL无水乙醇,充分振荡超声后置于油浴锅中,注入40 mL聚乙烯亚胺水溶液后,于55 ℃冷凝回流反应24 h。待反应结束后,将材料依次使用50 mL超纯水、丙酮、无水乙醚洗涤,回收反应后硅球材料置于60 ℃真空干燥箱中干燥12 h,备用,将材料标记为720 nm-NH_2_-GPTS-SiO_2_。

720 nm-NH_2_-GPTS-SiO_2_的硬质酰氯修饰:称取1 g上述反应制备的720 nm-NH_2_-GPTS-SiO_2_材料,加入150 mL双颈烧瓶中,通氮气后用橡胶塞封口,采用注射器注入10 mL二氯甲烷,超声使其分散均匀。配制0.1 g/mL硬质酰氯溶液(称取硬质酰氯固体1 g,溶于10 mL二氯甲烷),置于10 mL恒压滴液漏斗中,调整流速逐滴滴入双颈烧瓶中,冰水浴下过夜反应12 h。待反应结束后,将材料依次使用50 mL二氯甲烷、无水乙醇洗涤,置于60 ℃真空干燥箱中干燥,将材料标记为720 nm-C_18_-NH_2_-GPTS-SiO_2_。

采用相同的表面结构修饰方案修饰3 μm PS微球用作后续色谱性能对比,各步反应修饰产物分别记为3 μm-activated-SiO_2_、3 μm-GPTS-SiO_2_、3 μm-NH_2_-GPTS-SiO_2_、3 μm-C_18_-NH_2_-GPTS-SiO_2_。采用文献^[3]^相同方法在同款720 nm NPS微球表面修饰C_18_基团,记为720 nm-C_18_-SiO_2_。

### 1.3 毛细管色谱柱的制备

采用高压匀浆法将720 nm-C_18_-NH_2_-GPTS-SiO_2_色谱填料分别填入毛细管色谱填充柱。毛细管内径:100 μm,有效长度:10 cm,毛细管总长:30 cm。分别在毛细管填充柱末端柱塞后1 mm处烧制紫外检测窗口,用于后续pCEC平台色谱性能实验。

### 1.4 色谱分离条件

苯系物的分离:流动相A为10 mmol/L甲酸铵水溶液,甲酸调节pH至4.00;流动相B为乙腈。毛细管进口端接地,出口端施加+15 kv的电压,分流比为2000∶1,定量环1 μL,紫外检测波长214 nm,泵流速0.035 mL/min。梯度洗脱程序为0~10 min, 30%B~70%B; 10~40 min, 70%B。

多环芳烃化合物的分离:流动相A为10 mmol/L甲酸铵水溶液,甲酸调节pH至3.00;流动相B为乙腈。紫外检测波长254 nm。梯度洗脱程序为0~5 min, 45%B~70%B; 5~40 min, 70%B。其余条件同苯系物的分离。

## 2 结果与讨论

### 2.1 NPS微球与PS微球形态和表面结构的比较

由于制备方法的区别^[13]^, NPS微球与PS微球在比表面积、孔径孔容、表面硅羟基状态等方面存在差异,导致在其表面进行修饰反应及后续分离应用会产生较大不同。本研究以720 nm无孔二氧化硅微球(720 nm-NPS)与3 μm多孔二氧化硅微球(3 μm-PS)为研究对象,采用动态光散射法(DLS)测定两种微球的粒径信息,通过BET比表面积测试法对比二者的孔径、比表面积。并进一步采用傅里叶变换红外光谱法和600 MHz固体核磁共振波谱仪比较了两种二氧化硅微球表面羟基状态的区别。

2.1.1 孔结构的差异

DLS单分散性测试结果显示:两种二氧化硅微球的分散性均良好,其中3 μm-PS测得平均粒径为3056.63 nm,分散性系数为0.088; 720 nm-NPS平均粒径为718.94 nm,分散性系数为0.033。

如图1两种微球的氮气吸附与解析附曲线所示:3 μm-PS属于Ⅴ型吸附等温线,在中等压力处有回滞环的产生,体现氮气在微球的孔结构中有毛细凝聚的现象发生,符合多孔微球的特征;而在720 nm-NPS的吸附与解吸附等温线中,几乎没有回滞环的产生,属于Ⅲ型吸附等温线。通过进一步分析二者的孔径分布图可以看到,3 μm-PS除了存在大量2 nm以下的微孔以外,还有大量的10~20 nm的中孔(介孔)结构,而720 nm-NPS仅有极少量的2 nm以下的微孔,考虑微球间自身堆积产生孔结构的现象,可近似认为是无孔的,与文献^[14]^结果一致。同时PS微球的比表面积远大于NPS微球,二者的测试结果分别为317.6 m^2^/g和4.815 m^2^/g。

**图 1:**
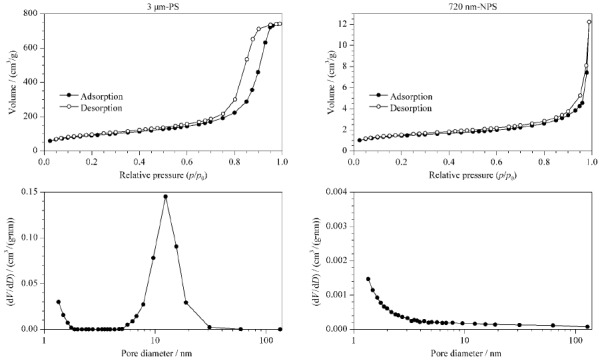
3 μm-PS与720 nm-NPS微球的N_2_吸附-解析附图和孔径分布图

2.1.2 硅羟基的差异

图2的红外图谱展示了未修饰的两种二氧化硅微球的官能团情况:3445 cm^-1^处的强宽峰一般来自于红外压片检测过程中所使用的溴化钾晶体所带结晶水中-OH的反对称伸缩振动峰,1637 cm^-1^处的峰同样来自样品中水的H-O-H弯曲振动峰;而1100 cm^-1^处宽而强的特征吸收峰为Si-O-Si的反对称伸缩振动峰,800 cm^-1^处和470 cm^-1^处的峰分别为Si-O-Si的对称伸缩振动峰和弯曲振动峰。

**图 2:**
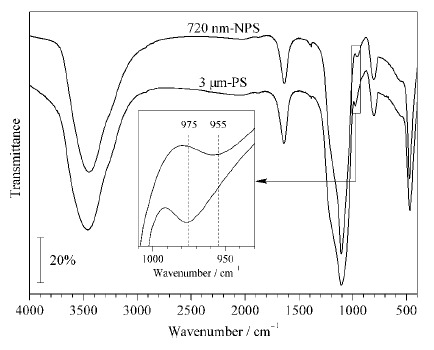
720 nm-NPS与3 μm-PS微球的红外光谱图

除了这些特征峰之外,本研究发现3 μm-PS的硅羟基出峰位置为975 cm^-1^,而720 nm-NPS的硅羟基出峰位置为955 cm^-1^,出现了微小程度的红移。该差异在本实验室自行合成或采购的其他品牌商品二氧化硅材料中均存在。NPS及PS微球的硅羟基红外峰存在20 cm^-1^的差异,可能是NPS及PS材料表面硅羟基的结构差异导致的不同。

二氧化硅微球表面的硅羟基及硅烷表面化学结构种类如图3所示可分为以下几类:表面硅氧烷(Q_4_)、孤立硅羟基(Q_3_)、偕硅羟基(Q_2_)以及当羟基与邻位的硅氧形成氢键时产生的邻硅羟基结构^[15]^。

**图 3:**
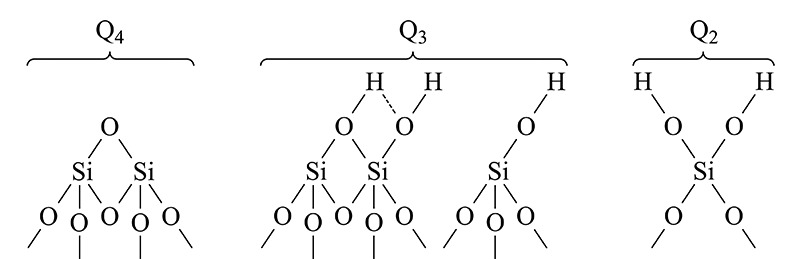
表面硅氧烷、邻位硅羟基、孤立硅羟基和偕硅羟基示意图

本研究进一步采用600 MHz固体核磁共振波谱仪检测^29^Si的核磁共振波谱来揭示NPS及PS材料表面硅羟基的结构差异。图4展示了720 nm-NPS与3 μm-PS在^29^Si核磁共振谱中的差异,*δ* 110处最强的峰为Si-O-Si硅氧烷的吸收峰(Q_4_); *δ* 100处为邻位硅羟基或是孤立的硅羟基(Q_3_); *δ* 90处则为偕硅羟基(Q_2_)的核磁共振峰。通过对核磁图谱解卷积并以面积归一化法定量分析:PS微球16%的硅原子带有一个硅羟基(孤立硅羟基,Q_3_)、19%带有两个硅羟基(偕硅羟基,Q_2_);而NPS微球不存在偕硅羟基,仅有30%硅原子处于孤立硅羟基状态。这进一步证明了红外图谱20 nm的差异是因为NPS及PS材料表面的硅羟基处于不同的状态。

**图 4:**
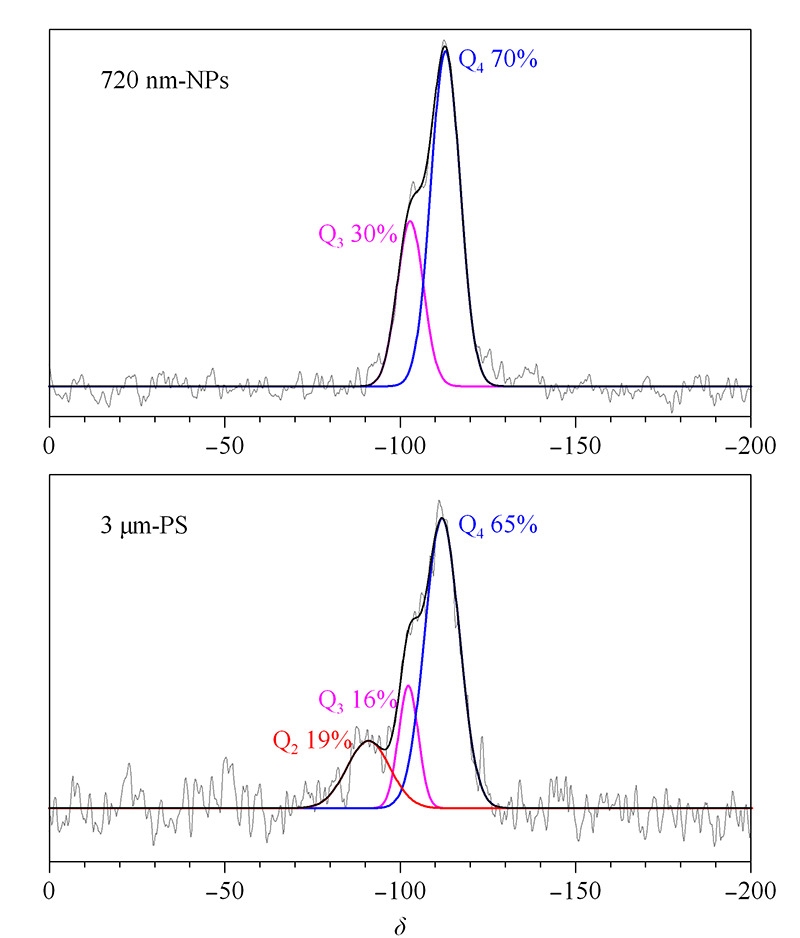
720 nm-NPS与3 μm-PS微球的^29^Si固体核磁图谱

可通过计算二氧化硅微球的总硅羟基比值(2Q_2_+Q_3_)/(Q_2_+Q_3_+Q_4_)进一步对比硅羟基的数量^[16]^: NPS微球的总硅羟基比值为0.30,小于PS微球的0.54。而且偕硅羟基也在文献^[17]^中被证实表面修饰活性远超于孤立硅羟基和邻位硅羟基,因此这也解释了亚微米NPS微球表面修饰反应活性较低,难以用常规十八烷基氯硅烷等简单一步反应修饰得到高的碳含量。鉴于无孔和多孔表面羟基结构的差异主要来源于两种基质材料本身的合成方式的不同,所以本文后续采用多步反应利用聚合物增加反应位点,制备高碳含量的新型亚微米NPS微球。

### 2.2 高碳含量二氧化硅微球的合成与表征

高碳含量NPS微球的具体合成过程如图5所示:将酸活化后的SiO_2_用GPTS硅烷化试剂修饰得到具有环氧丙基的GPTS-SiO_2_,利用环氧丙基与氨基的共价键反应将聚乙烯亚胺包覆在硅球表面得到NH_2_-GPTS-SiO_2_,最后以硬脂酰氯酰基化修饰得到具有长碳链的C_18_-NH_2_-GPTS-SiO_2_。该反应的特点在于PEI聚合物的引入,利用PEI自身具有大量氨基反应位点的特性来提高后续碳的含量。

**图5:**
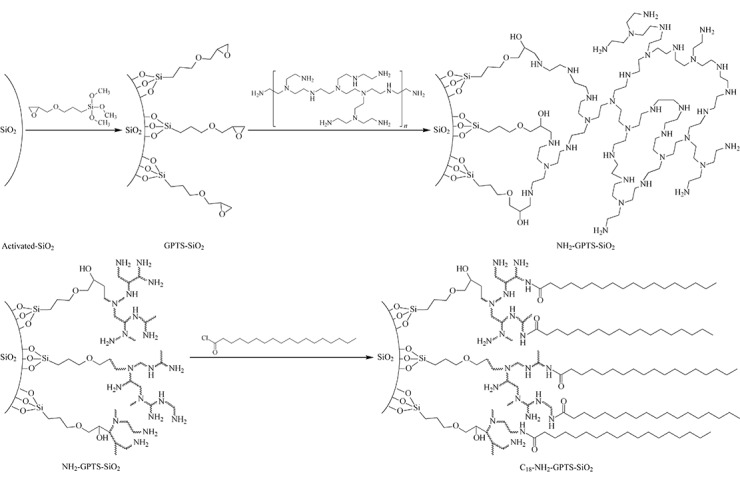
高碳含量NPS微球表面结构修饰制备过程示意图

2.2.1 元素分析

采用元素分析对修饰过程中各步反应的氮、碳、氢3种元素的含量变化进行监测。如表1所示:720 nm的NPS微球碳含量从0.55%增加到了8.29%; 3 μm的PS微球碳含量从0.34%增加到了20.15%。如2.1节中所提到的,NPS微球由于比表面积低及硅羟基种类数量的限制,在该修饰方案下碳含量增幅不如PS微球,但8.29%的数据已远高于采用传统C_18_修饰方案修饰的同款亚微米NPS材料0.79%的数据,说明该C_18_-NH_2_-GPTS-SiO_2_修饰方法已经很成功地实现了提高亚微米NPS微球表面碳含量的目的。

**表 1:** 结构修饰过程元素分析结果

Sample	N/%	C/%	H/%
720 nm-activated-SiO_2_	0.13	0.55	0.99
720 nm-GPTS-SiO_2_	0.16	1.99	1.23
720 nm-NH_2_-GPTS-SiO_2_	0.31	2.19	1.30
720 nm-C_18_-NH_2_-GPTS-SiO_2_	0.27	8.29	2.34
3 μm-activated-SiO_2_	< 0.10	0.34	0.61
3 μm-GPTS-SiO_2_	< 0.10	7.73	1.50
3 μm-NH_2_-GPTS-SiO_2_	2.70	9.59	2.29
3 μm-C_18_-NH_2_-GPTS-SiO_2_	2.20	20.15	3.56
720 nm-C_18_-SiO_2_	<0.10	0.79	0.54

2.2.2 傅里叶变换红外光谱

FT-IR中3000 cm^-1^以下的饱和碳氢红外吸收峰可用于表征亚微米NPS微球修饰过程中的各步反应。图6a中GPTS-SiO_2_的谱带相比未修饰硅球多了2920 cm^-1^和2850 cm^-1^处的吸收峰,分别表示-CH_2_的反对称伸缩振动峰和对称伸缩振动峰,这表明了GPTS的成功修饰。而在硬脂酰氯修饰后的C_18_-NH_2_-GPTS-SiO_2_谱带中,2960 cm^-1^处代表-CH_3_基团的吸收峰相对-CH_2_吸收峰较小,这与硬质酰氯中-CH_3_与-CH_2_基团的比例近似,因此印证了C_18_基团的成功修饰。

**图 6:**
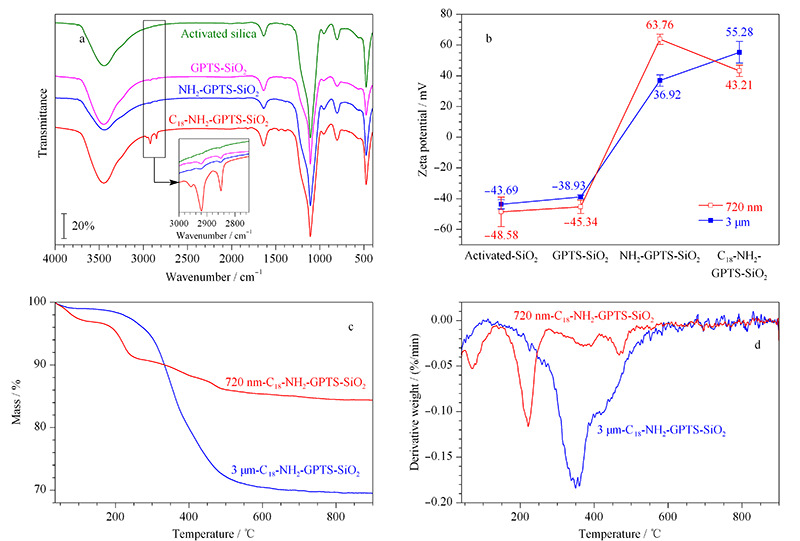
表面结构修饰过程的(a)红外光谱图(720 nm)、(b)Zeta电势、(c)TGA和(d)热重差值(DTG)曲线

2.2.3 Zeta电势

红外图谱很难展现出聚乙烯亚胺包覆前后官能团的差异,本研究采用Zeta电势来辅助验证这一修饰过程,通过将各反应产物在无水乙醇中配制成0.3 mg/mL的混悬液,得到如图6b所示结果:经酸活化的二氧化硅微球及GPTS修饰后的微球由于表面带有大部分硅羟基,其表面Zeta电势为负值,而在聚乙烯亚胺包覆后Zeta电势发生了电性的反转,从表面带羟基的负电势状态增大到正值,该过程补充证明GPTS-SiO_2_与NH_2_-GPTS-SiO_2_红外图谱差异不大的现象,说明了经过聚乙烯亚胺的包覆后,二氧化硅微球表面主要带氨基,为正电荷。在硬脂酰氯的修饰反应中,720 nm-C_18_-NH_2_-GPTS-SiO_2_的Zeta电势略有降低,而3 μm C_18_-NH_2_-GPTS-SiO_2_的Zeta电势继续升高,体现出氨基包覆与多孔材料的结合更为紧密,聚合物结合在微球内部,不易因后续反应失去。

2.2.4 热重分析

图6c展示了C_18_-NH_2_-GPTS-SiO_2_修饰方案修饰后的720 nm与3 μm SiO_2_微球的TGA曲线。3 μm SiO_2_微球的质量损失较720 nm微球大得多,该结果与元素分析中两者碳含量差异(20.15%和8.29%)的结果相符。TGA曲线微分后得到的DTG曲线(见图6d)可以分析2种材料的不同质量损失阶段,720 nm微球的质量损失可以明显地被分成4个阶段:第一个阶段发生在100 ℃以下,可认作是材料表面吸附水的流失和吸附气体的挥发;第二个阶段发生在210 ℃附近,其质量损失幅度最大,可归结为C_18_-NH_2_-GPTS-SiO_2_修饰方案中最后一步通过硬质酰氯修饰提供的C_18_碳链的流失,同样符合元素分析结果中720 nm NH_2_-GPTS-SiO_2_微球与720 nm C_18_-NH_2_-GPTS-SiO_2_微球碳含量从2.19%到8.29%的最大增长;第三个阶段发生在390 ℃左右,可以认为是材料表面包覆的聚乙烯亚胺聚合物的燃烧,因为大分子聚合物的燃点较之小分子化合物更高;第四个阶段发生在460 ℃附近,其可认为是硅球表面最后包覆的一层GPTS硅烷化试剂。反观3 μm PS微球的DTG微分曲线,其几个质量损失阶段有所重合,第一阶段100 ℃以下,材料表面吸附水的流失和吸附气体的挥发过程与720 nm SiO_2_微球相仿,最大的差别来自于第二个阶段C_18_碳链的流失较720 nm SiO_2_微球的流失温度更高,与第三、第四个阶段有所重合,显示C_18_碳链与3 μm SiO_2_微球结合更为紧密。考虑到其具有多孔结构,碳链修饰在微球内部,所需质量流失的温度也更高。

2.2.5 扫描电镜

图7展示了720 nm和3 μm SiO_2_微球在C_18_-NH_2_-GPTS-SiO_2_修饰方案修饰后的形貌变化,相较于修饰前较为光滑的表面,在修饰后的微球表面较为粗糙,具有一层表面结构修饰的包覆层。

**图 7:**
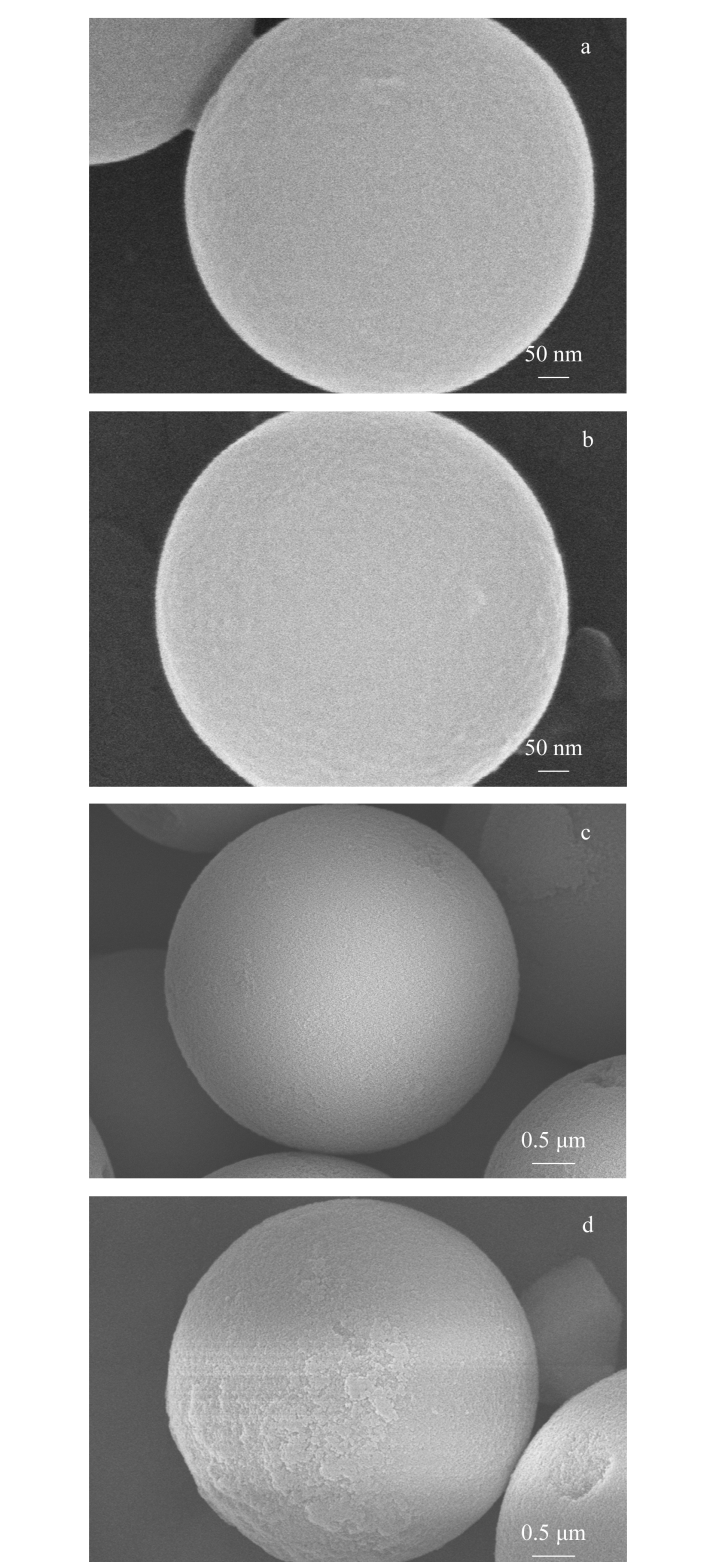
二氧化硅微球表面结构修饰前、后的SEM照片

### 2.3 C_18_-NH_2_-GPTS-SiO_2_色谱填料在毛细管电色谱平台的机理考察

2.3.1 EOF的比较

图8a展示了720 nm C_18_-NH_2_-GPTS-SiO_2_与3 μm C_18_-NH_2_-GPTS-SiO_2_材料毛细管填充柱在不同pH值、不同电压下EOF的变化情况。720 nm C_18_-NH_2_-GPTS-SiO_2_色谱柱的EOF对pH值变化敏感,电渗流随着pH值的减小而急剧增大,其特性应与该种材料表面的基团有关。从红外与Zeta电势的实验结果中可以看到,改性后的硅球表面除了含有大量的氨基使得微球表面带正电荷外,从红外图谱上还可以观察到存在部分残留羟基(955 cm^-1^处),其很可能是反应活性较低未与GPTS反应的孤立硅羟基,两种基团的同时存在导致该材料需在酸性pH应用以获取极快的电渗流。而3 μm C_18_-NH_2_-GPTS-SiO_2_材料毛细管填充柱,由于该大颗粒多孔材料表面多是反应活性高的偕硅羟基,其在本修饰方法中已被完全取代,材料表面仅存带正电荷的氨基,故其EOF对pH值相对不敏感。

**图 8:**
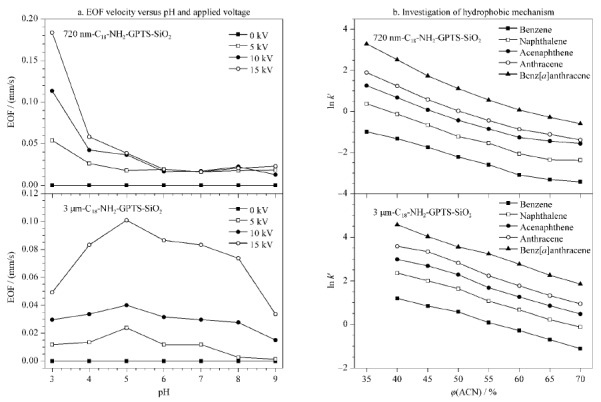
720 nm与3 μm C_18_-NH_2_-GPTS-SiO_2_色谱柱的(a)电渗流随pH、施加电压的变化关系和(b)反相作用机理考察

2.3.2 C_18_-NH_2_-GPTS-SiO_2_色谱填料反相机理的考察

图8b比较了苯、萘、苊、蒽和苯并[*a*]蒽等5种物质的调整保留因子随流动相中乙腈体积分数的变化关系,可以观察到:无论是3 μm还是720 nm的C_18_-NH_2_-GPTS-SiO_2_毛细管填充柱均符合反相色谱机理,苯、萘、苊、蒽和苯并[*a*]蒽等无机物小分子的保留时间均随着流动相中乙腈体积分数的增加而降低。可见该表面结构修饰方案除了通过氨基包覆的方式加快电渗流之外,还以硬质酰氯修饰提供长碳链的方式提高亚微米材料表面碳含量,提高反相机理下的分离能力,具有实际意义,且本文的合成方法相较于采用“树枝状”或是“刷子型”^[9,18]^等其他提高碳含量的修饰方法,具有反应相对简单、可以一次性利用高分子聚合物提供更多反应位点的优势。

### 2.4 C_18_-NH_2_-GPTS-SiO_2_材料色谱分离性能评价

2.4.1 苯系物的分析

图9a对比了在pCEC模式下,施加不同电压对于6种苯系物分离效果的变化,可以观察到在pH值为4时,相比微径液相模式,施加电压能显著提高线性流速,缩短分析时间,且随着施加电压的增高峰形有一定的改善。

**图 9:**
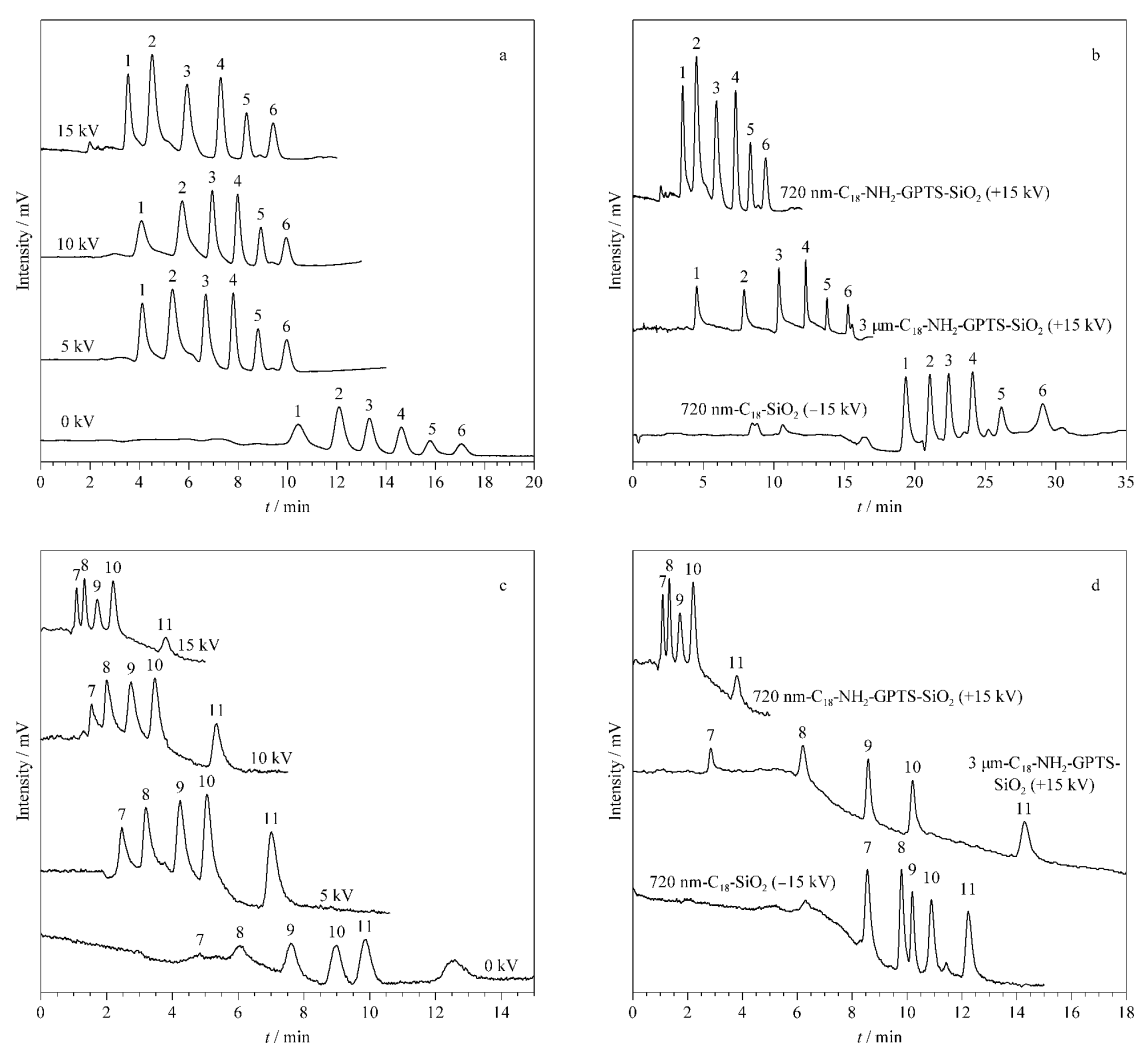
施加电压对720 nm C_18_-NH_2_-GPTS-SiO_2_色谱柱分离(a)苯系物与(b)多环芳烃的影响和不同色谱柱分离(c)苯系物与(d)多环芳烃效果对比

图9b则对比了720 nm C_18_-NH_2_-GPTS-SiO_2_、3 μm C_18_-NH_2_-GPTS-SiO_2_毛细管填充柱和传统方法制备的720 nm C_18_-SiO_2_毛细管填充柱之间在同一色谱条件下的分离效果对比。传统方法制备的720 nm C_18_-SiO_2_毛细管填充柱表面带羟基,为负电荷,与前两种氨基修饰带正电荷相反,故分离时采用在毛细管后端施加-15 kV的电压以获得同样正向的电渗流。从结果中可以看到,采用本修饰方案的720 nm和3 μm C_18_-NH_2_-GPTS-SiO_2_毛细管填充柱在分离速度有明显优势,说明通过将表面改为大量氨基的方法可以获得更高的电渗流,使pCEC分离过程中的线性流速增加,从而缩短分析时间。

2.4.2 多环芳烃类化合物的分析

图9c对比了在pCEC模式下,施加不同电压对5种多环芳烃类化合物分离效果的影响。与图8a中EOF的实验结果相印证的是,在pH为3的条件下,电渗流的大小随施加电压的增加呈倍数增长,此时相比压力流,电渗流的大小对色谱分离速度起到主导作用,720 nm C_18_-NH_2_-GPTS-SiO_2_色谱填充柱中各组分的分离速度随着施加电压的增加而大幅加快,在+15 kV的条件下达到最高线性流速,实现了在5 min内对5种化合物的快速分离。

图9d同样对比了多环芳烃类化合物在3种色谱填充柱的分离效果:与2.4.1中的实验结果相似,采用本修饰方案的720 nm C_18_-NH_2_-GPTS-SiO_2_毛细管填充柱在分离速度的优势上最为明显,分离速度明显大于3 μm C_18_-NH_2_-GPTS-SiO_2_毛细管填充柱和传统720 nm C_18_- SiO_2_毛细管填充柱。而且由于碳含量的增加,分离效果上也比传统C_18_修饰色谱柱更佳。由此可见,720 nm C_18_-NH_2_-GPTS-SiO_2_毛细管填充柱具有两大优势:其一是通过氨基的包覆将二氧化硅微球表面的电性改变,并大幅加快电渗流以提高分离速度;其二是通过酰胺键将长碳链与氨基连接,提高亚微米无孔材料的碳含量,在反相机理下取得更佳的分离效果。

## 3 结论

本文利用pCEC平台的优势,将压力流与电渗流作为共同驱动力,规避了亚微米NPS微球在传统液相色谱上应用时反压过大的限制^[19]^。本文针对亚微米NPS微球表面性质进行深入探究,发现了NPS材料具有表面硅羟基相对较少,缺少高反应活性的偕硅羟基,表面修饰困难的特征,并针对这一特点,设计了一种具有高碳含量的新型表面结构修饰方法。该方法创新性地利用聚合物包覆的方式提供了更多的反应位点,使得亚微米NPS微球表面碳含量大幅提高,并且在pCEC平台应用时,氨基的包覆使得电渗流大幅加快,提高了色谱分离速度。可见C_18_-NH_2_-GPTS-SiO_2_作为一种非常适用于pCEC平台应用的优异材料修饰方法,为pCEC平台色谱材料的开发与亚微米NPS微球的应用提供了新的思路和参考。
